# Partitioning diversity in subterranean invertebrates: The epikarst fauna of Slovenia

**DOI:** 10.1371/journal.pone.0195991

**Published:** 2018-05-02

**Authors:** Tanja Pipan, David C. Culver, Federica Papi, Peter Kozel

**Affiliations:** 1 Karst Research Institute of the Scientific Research Centre of the Slovenian Academy of Sciences and Arts, Postojna, Slovenia; 2 Karstology Study Program, University of Nova Gorica, Nova Gorica, Slovenia; 3 Department of Environmental Science, American University, Washington, District of Columbia, United States of America; Universidade Federal de Mato Grosso do Sul, BRAZIL

## Abstract

The decomposition of diversity into within site (α) and between site (β) components is especially interesting in subterranean communities because of their isolated nature and limited dispersal potential The aquatic epikarst fauna, sampled from water drips in caves affords a unique opportunity to provide comparable, quantitative samples of a portion of the obligate subterranean dwelling fauna in multiple hierarchical levels. We focused on three interrelated questions—(1) what is the spatial pattern of epikarst species diversity; (2) how does species diversity partition between local, and regional components (nested and replacement); and (3) whether epikarst hotspots are subterranean hotspots in general. We analyzed the geographic pattern of species richness of 30 species of obligate subterranean copepods found in 81 drips in Slovenian caves in three karst regions—Alpine, Dinaric, and Isolated. Comparison of Chao1 and observed (Mao-tau) estimates of species richness indicated sampling in most drips was complete, but species accumulation curves indicated roughly half of the sites in the Dinaric karst had not reached an asymptote. Overall, within drip diversity accounted for three species, different drips in a cave another three, different caves in a region six species, and different regions accounted for the remaining 18 species. Sites in the Dinaric karst had much higher species richness than the other sites, which is in agreement with studies of other components of the subterranean fauna. The fauna associated with drips in Županova jama (jama = cave), in the east-central Dinaric karst was the richest found. While turnover explained the majority of β-diversity, nestedness in the form of hotspot drips was important as well. A consequence is that a small number of drips largely determine cave and regional species diversity.

## Introduction

It has long been recognized, at least since 1960 [1], that the regional biodiveristy (γ) can be decomposed into local (α) and between site (β) diversity. MacArthur [2] equated within-habitat diversity to α-diversity and between habitat diversity to β-diversity, but more generally β-diversity has come to mean between site diversity, even if the habitats are quite similar. Partitioning of diversity can be extended across multiple hierarchical scales to determine the contribution of each level to total richness [3]. Baselga [4,5] made the important distinction between β-diversity due to replacement of one species by another in the region, and nestedness, where the poorest assemblage is a strict subset of the richest one. Beta-diversity is a useful way to understand species diversity in cave fauna. While caves differ in many aspects one from the others, more than most habitats, they can be considered replicate habitats due to the absence of light and scarce organic matter [6]. Since movement between caves by species limited to subterranean habitats is highly restricted [7], β-diversity should largely be due to replacement, and because migration is highly restricted between different cave regions in different karst areas, it should be especially pronounced at larger geographic scales. This has largely been confirmed in two European studies of the aquatic cave fauna [8,9].

However, the global and continental patterns of species richness of the subterranean fauna are quite different from those of most biota [10,11,12]. Hotspots of α-diversity (species-rich individual caves) are mostly in North Temperate areas rather than in tropical areas [12,13], but all regions have terrestrial and aquatic species that are limited to caves and other subterranean habitats. The commonly held view is that climate changes in the temperate zone, including, but not limited to the Pleistocene, were forcing agents for colonization of subterranean habitats [6,7,10,11]. However, even glaciated regions have a few cave-limited aquatic species (stygobionts) that survived glaciation by living under the ice sheets [14]. Detailed regional studies of α- and β-diversity of European and North American cave fauna [8,11,15,16] indicate that the Dinaric karst, extending from northeast Italy to the Albanian coast, is a global hotspot of both aquatic and terrestrial subterranean biodiversity.

Within these overarching patterns, there is often considerable lumpiness in maps of subterranean species richness, with hotspots extending only a few tens of kilometers. In Deharveng et al.’s [15] map of aquatic subterranean species richness at a grid scale of 0.2^o^ (312–403 km^2^), hotspots (variously defined) were almost never larger than three or four units (about 1600 km^2^). In a detailed study of the troglobiotic (cave-limited) beetle fauna of the Dinaric karst that corrected for differences in collecting intensity, Zagmajster et al. [16] found that hotspot areas were about 20 X 20 km.

Beyond these broad-brush strokes of the pattern of subterranean biodiversity, there is considerable uncertainty. It is well known that data are incomplete, and that most caves in any area (especially the smaller caves), even well sampled ones like Slovenia or eastern North America, have not been studied [10]. Because β-diversity is typically much higher than α-diversity in subterranean communities [8,9], incomplete sampling results in missing species, especially if most β-diversity is due to replacement. Finally, caves are generally difficult to access and sample, and not all species that are present are seen in a single census, resulting in false negatives [17]. There are techniques available to estimate all or part of the ‘missing’ species richness [18], but their application to cave and other subterranean databases requires large amounts of data, which are rarely available (but see [16]).

The alternative is to use a sampling scheme that is quantitative, replicable, and can be assessed for its completeness. In karst regions, an unbiased, quantitative sampling techniques is the collection of epikarst fauna by the continuous filtering of drip water in caves, water that is exiting the epikarst [19]. Epikarst is the boundary region between soil and rock in karst, usually honeycombed with small fractures, solution pockets, and solutionally widened trenches [20,21,22]. The epikarst is organized into a series of small drainage basins, with a catchment area of between 0.1 and 300 m^2^ [23], at least an order of magnitude less than that of a cave. The invertebrate inhabitants can be dislodged by the current [24] or drift into dripping water which can be periodically filtered for the dislodged invertebrates. If sampling continues for a year, then the accumulation curves of species richness reach an asymptote, in the majority of cases [25]. While the sampling is indirect, it is highly repeatable. Because sampling is from a single drip, and multiple drips are typically sampled within a cave, the data naturally clusters into three scales. The finest scale is the individual drip; the second is that of a quadrat 1 km^2^ in size. This quadrat covers all or nearly all of the drip samples within a cave, at least in Slovenian studies (e.g. [19]). Informally, drips can be grouped by the cave in which they are found, but the epikarst fauna has no direct connection with caves other than that the sampling point is in a cave. Because caves and cave passages only occur in a few quadrats of this size in a karst area, most quadrats cannot be sampled. Elements of this epikarst fauna can also be collected in drip pools in caves [26], but this is a biased sample contaminated with more widespread generalist species [27]. The third scale is that of karst areas that share a common geological history, blocks of continuous limestone typically extending tens to hundreds of kilometers in linear extent [28]. Thus, the scale of an individual habitat (α-diversity) is approximately 100 m^2^, that of the cave (or quadrat) is 1 km^2^ and that of a region approximately 1000 km^2^.

In this contribution we examine the geographic pattern of species richness for intensively sampled drips in karst areas in Slovenia. Data from continuous sampling of epikarst drips make it possible to ask several questions about the geography of epikarst community richness, makes the answers less susceptible to change with increased sampling. The first of these is just what is the spatial pattern of epikarst species richness in Slovenia? Are there regional patterns among different karst areas as well as local patterns? The second is what is the partition of species richness into (α-diversity) and β-diversity, and the partition of β-diversity into replacement and nestedness components? The third is whether epikarst hotspots correspond to cave hotspots?

## Materials and methods

### Study area

**Fig 1:**
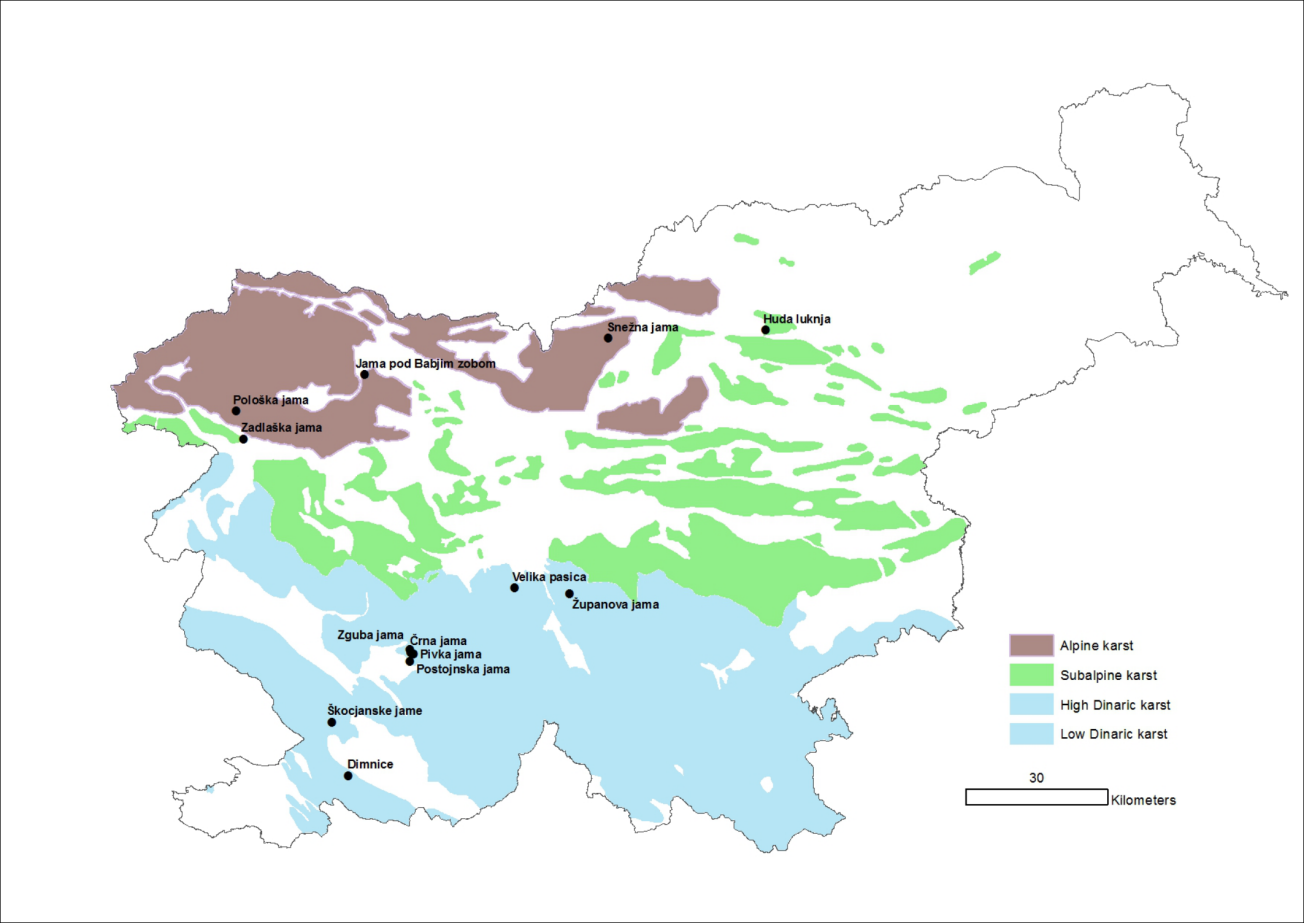
Map of Slovenia with its four karst regions (from [31]) along with sampling sites. Since the High Dinaric karst and the Low Dinaric karst are intercalated, are typically treated as a single unit. We treat it as a single unit here. Modified from Gams [33].

Slovenia is one of the most karstified countries in the world, with almost half of its land area covered by karst landscapes, with more than 10,000 known caves [29]. There are three main karst regions—Dinaric, Isolated, and Alpine (Fig 1)—and a total of 81 drips have been sampled for one year, accessed from 13 caves. Seventeen drips in two caves were sampled in the Isolated karst—Huda luknja [30] and Zadlaška jama [31]; 15 drips in three caves in the Alpine karst (Pološka jama, Jam pod Babjim zobom, and Snežna jama) were sampled by Papi in her unpublished dissertation [31]; and 49 drips in eight caves in the Dinaric karst were sampled. The Dinaric caves sampled included those in Pipan’s unpublished thesis [32]—Črna jama, Dimnice, Pivka jama, Postojnska jama, Škocjanske jame, and Županova jama; Velika pasica [26], and unpublished data of Kozel and Pipan on Zguba jama. Sampling length and number of drips varied among caves (Table 1). In all caves, all drips in a cave were within 900 m of each other, hence they could all be covered by a 1 km^2^ quadrat. Closest drips in nearby caves (Črna jama, Pivka jama, and Postojnska jama) were all greater than 1000 m apart. The actual position of drips was controlled by cave morphology, and in general, all drips in a passage segment were utilized.

**Table 1:** List of Slovenian caves sampled for epikarst copepods, number of drips, average number of samples taken, sampling start date, and karst region (defined in [33]). Sampling was typically done at monthly intervals.

Cave	Elevation (m)	N drips	N samples/ drip	Max. linear distance between points	Start date of sampling	Karst region
Črna jama	540	5	5	150	10/2000	Dinaric
Dimnice	567	5	9	250	5/2000	Dinaric
Huda luknja	508	12	12	175	11/2005	Isolated
Jama pod Babjim zobom	860	5	8	200	8/2007	Alpine
Pivka jama	540	5	5	170	10/2000	Dinaric
Pološka jama	730	5	8	900	12/2006	Alpine
Postojnska jama	529	10	7	200	4/2000	Dinaric
Snežna jama	1556	5	7	300	9/2006	Alpine
Škocjanske jame	425	5	9	850	5/2000	Dinaric
Velika pasica	670	4	88	40	5/2006	Dinaric
Zadlaška jama	298	5	7	70	10/2006	Isolated
Zguba jama	561	10	12	80	4/2012	Dinaric
Županova jama	468	5	9	150	5/2000	Dinaric

### Fauna

Samples were collected with a funnel in a continuous filtering device (described in [19]) and removed at monthly intervals for sorting and identification. Samples were preserved in alcohol. Monthly sampling minimized the effects of predation within the samples, and provided the benefit of repeated samples at each drip. Only copepods were used in this analysis, but they represent the large majority of aquatic crustaceans and other invertebrates present in the samples [19,30]. Only obligate epikarst subterranean dwelling species (stygobionts), both described and undescribed, are included in the analysis.

### Data analysis

Species richness and its standard error were estimated using the individual based Mao-tau analytical function [34], and the Chao1 estimate of total richness [35], using EstimateS 9.1 [18], and calculated with the formulas

$$\begin{array}{l} S_{Chao1}=S_{obs}+[\left(n-1\right)/n][F_{1}^{2}/{2F}_{2}]\;(unbiased)\\ \;S_{Chao1}=S_{obs}+[F_{1}^{2}/{2F}_{2}]\;(classic) \end{array}$$

where S_obs_ was the observed number of species, n the number of samples, and F_i_ the number of species with exactly i individuals. These formulas correct for collecting intensity. Previous analyses of accumulation curves for some of the data from the Dinaric karst, indicated that the number of samples taken was sufficient to uncover most or all of the species [25], and this was often the case for the larger data set analyzed here. Mao-tau and Chao1 estimates and number of samples were uncorrelated for each region, and so extrapolation or truncation of data were not used. As recommended in EstimateS [18], the classic formula was only used when the coefficient of variation of abundance was greater than 50%. At those cases, the larger of the ACE and Chao1 estimate was used. For aggregated estimates for karst regions, incidence based Chao2 and ICE (Incidence Coverage Estimates) were used, using the following formulas for ICE:

$$\begin{array}{l} S_{ice}=S_{freq}+\left[S_{infr}/C_{ice}\right]+\left[\left(Q_{1}/C_{ice}\right)\Upsilon_{ice}^{2}\right]\;where\\ C_{ice}=1-[Q_{1}/N_{infr}]\\ N_{infr}=\sum_{j=1}^{10}{j\;Q}_{j}\\ \;\Upsilon^{2}=max\left\{[\left(S_{infr}/C_{ice})(m_{infr}/m_{infr-1})({\sum_{j=1}^{10}{j(j-1)Q}_{j}/\left(N_{infr}\right)}^{2}\right)]-1,0\right\} \end{array}$$

where Q_1_ is the number of singletons, S_freq_ is the number of species in more than 10 samples, S_infr_ the number of species in 10 or fewer samples, Υ^2^_ice_ the coefficient of variation for Q_i_ for infrequent species, m_infr_ the number of samples with at least one infrequent species, and N_infr_ the number of occurrences of infrequent species. Incidence curves were also computed, but extrapolations [18] were not.

Species diversity was partitioned into within drips, among drips, among caves, and among regions using Partition 3.0 [36]. β-diversity was decomposed by the following [4, 37]

$$\beta_{sor}=\beta_{sim}+\beta_{sne}=\left(b+c\right)/\left(2a+b+c\right)=[b/\left(b+a\right)]+[\left(c-b\right)/\left(2a+b+c\right)][a/\left(b+a\right)]$$

where β_sor_ is Sorenson dissimilarity, β_sim_ is Simpson dissimilarity (= replacement), β_sne_ is the nestedness component, a is the number of shared species, b is the number of species unique to the poorer site and c the number of species unique to the richer site. This decomposition was computed for among drips within a cave, among caves within a region, and among regions using betapart, an R package [36]. The expectation under the Null hypothesis was generated using individual randomizations.

The Kruskal-Wallis test, a non-parametric analog to a one-way ANOVA was used to compare regions, in order to avoid the assumption of normality of species counts, which were generally not normally distributed, according to cumulative frequency plots. Mean species number and maximum species number per drip were compared by regression analysis on number of species per cave. Accumulation curves, with caves as the sampling unit, were compared for the Dinaric karst and the Alpine karst, the two regions with more than two sampled caves.

Mapping of locations was done using ArcMap^TM^ 10.3.1. Cave locations and shapefiles of karst areas were provided by the Karst Research Institute ZRC SAZU. Basic statistics were computed in Excel^TM^ and JMP^TM^.

## Results

### Spatial pattern of epikarst species richness

A total of 30 species were found in the 81 drips sampled in 13 caves (Table 2). *Speocyclops infernus* was found in all caves except Huda luknja; no other species was found in more than 8 caves. Half (15 of 30) species were known from a single cave, typically from only one or two drips in that cave. The number of species found in the caves ranged from 2 (Huda luknja and Snežna jama) to 13 (Županova jama) (Table 2). *S*. *infernus* also occurred in the largest total number of drips—30. *Parastenocaris nolli alpina* was the next most widespread species, occurring in 23 drips in 8 caves. Eight species were exclusively found in a single drip.

**Table 2:** List of stygobiotic copepod species found in the 81 drips in 13 caves in Slovenia, along with the number of drips and caves each species was found in.

Copepod Species	Črna jama	Dimnice	Huda luknja	Jama pod Babjim zobom	Pivka jama	Pološka jama	Postojnska jama	Snežna jama	Škocjanske jame	Velika pasica	Zadlaška jama	Zguba jama	Županova jama	N drips	N caves
**CYCLOPOIDA**															
*Diacyclops languidoides*													1	1	1
*Speocyclops infernus*	3	1		2	4	2	2	3	3	4	2	1	3	30	12
**HARPACTICOIDA**															
*Bryocamptus balcanicus*	2		2		1		1					4	3	13	6
*Bryocamptus pyrenaicus*										3			2	5	2
*Bryocamptus typhlops*										4				4	1
*Bryocamptus* n.sp. 1					1									1	1
*Bryocamptus* n.sp. 2						3		3			1			7	2
*Elaphoidella cvetkae*	2				4				1			2	3	12	5
*Elaphoidella kieferi*									2					2	1
*Elaphoidella stammeri*													2	2	1
*Elaphoidella millennii*										3			1	4	2
*Elaphoidella tarmani*										1				1	1
*Elaphoidella* n.sp. 1					2									2	1
*Elaphoidella* n.sp. 2				3										3	1
*Elaphoidella* n.sp. 3				1										1	1
*Lessinocamptus* n.sp.						5								5	1
*Marenobiotus* cf. *brucei*					2									2	1
*Moraria alpina*											1			1	1
*Moraria stankovitchi*													1	1	1
*Moraria* n.sp. 1	1								1					2	2
*Moraria* n.sp. 2													1	1	1
*Morariopsis dumonti*										4			1	5	2
*Morariopsis scotenophila*		1										2		3	2
*Nitocrella* n.sp.		2					1							3	2
*Parastenocaris* cf. *andreji*		1												1	1
*Parastenocaris nolli alpina*	1	2	3		4				4	1		3	5	23	8
*Parastenocaris* n.sp. 1	1	2												3	2
*Parastenocaris* n.sp. 2	3	2			4		1		3			3	1	17	7
*Parastenocaris* n.sp. 3									1				1	2	2
*Stygepactophanes*n.sp.	2	3							1					6	3
TOTAL SPECIES	8	8	2	3	8	3	4	2	8	7	3	6	13		

The richest cave (Chao1), is Županova jama, with 13 observed and predicted species (Table 3). All caves in the Dinaric karst had higher numbers of stygobiotic copepods than caves in either the Alpine karst or the Isolated karst (Fig 1), where each cave had three or fewer species. According to the non-parametric Kruskal-Wallis test, there were significant differences among regions (χ^2^ = 8.95, df = 2, p = .011). Based on Chao1 estimates, most species were found, except for Škocjanske jame, where the Chao1 estimate is three species higher than the observed number. In Pivka jama, Chao1 analysis predicted one additional species was present (Table 3).

**Table 3:** Epikarst copepod species richness in the 13 Slovenian study caves, ranked from highest to lowest species richness. Mao-tau estimates and standard errors are for observed numbers of species and Chao1 estimates include the likelihood of additional species based on the frequency of singleton species. Total N is the total number of copepods collected. See Fig 1 for regions.

			Mao tau S		Chao1			
Cave	Drips	Total N	Mean	SD	Mean	SD	N of samples	Region
Županova jama	Total	152	13	1.87	13.33	0.92	5	Dinaric
Škocjanske jame	Total	170	8	1.15	10.98	4.43	5	Dinaric
Pivka jama	Total	210	8	1.21	8.99	2.23	5	Dinaric
Dimnice	Total	35	8	0.25	8	0.24	5	Dinaric
Črna jama	Total	195	8	0.28	8	0	5	Dinaric
Velika pasica	Total	1287	7	0.82	7	0.36	4	Dinaric
Zguba jama	Total	396	6	0.43	6	0.09	10	Dinaric
Postojnska jama	Total	10	4	1.41	4.45	1.18	10	Dinaric
Jama pod Babjim zobom	Total	311	3	0	3	0	5	Alpine
Pološka jama	Total	42	3	0	3	0.04	5	Alpine
Zadlaška jama	Total	6	3	0	3	0	5	Isolated
Snežna jama	Total	188	2	0	2	0	5	Alpine
Huda luknja	Total	4	2	0	2	0	12	Isolated

There is considerable heterogeneity in the number of species among drips in each cave (Table 4). Of the 81 drips, 25 (31 percent) had no copepods at all. In contrast, 14 (17 percent) had at least 75 percent of the total number of species reported for that cave (quadrat). A total of 11 drips had 5 or more species, and all of these drips were in the Dinaric karst.

**Table 4:** Distribution of the number of species in the 81 drips sampled, arranged according to the cave in which they occur.

				Number of Species in Individual Drip								
	0	1	2	3	4	5	6	7	8	9	10	Cave Total
**Črna jama**			2	1		1	1					8
**Dimnice**		2	1			1		1				8
**Huda luknja**	9	2	1									2
**Jama pod Babjim zobom**	1	2		2								3
**Pivka jama**			1		3		1					8
**Pološka jama**		2	2	1								3
**Postojnska jama**	6	3	1									4
**Snežna jama**	2		3									2
**Škocjanske jame**		1		3		1						8
**Velika pasica**					2		2					7
**Zadlaška jama**	2	2	1									2
**Zguba jama**	5	1	1	1	2							6
**Županova jama**		1			1	2					1	13
**TOTAL**	25	16	13	8	8	5	4	1			1	30

### Partitioning of species diversity among hierarchical scales

**Fig 2:**
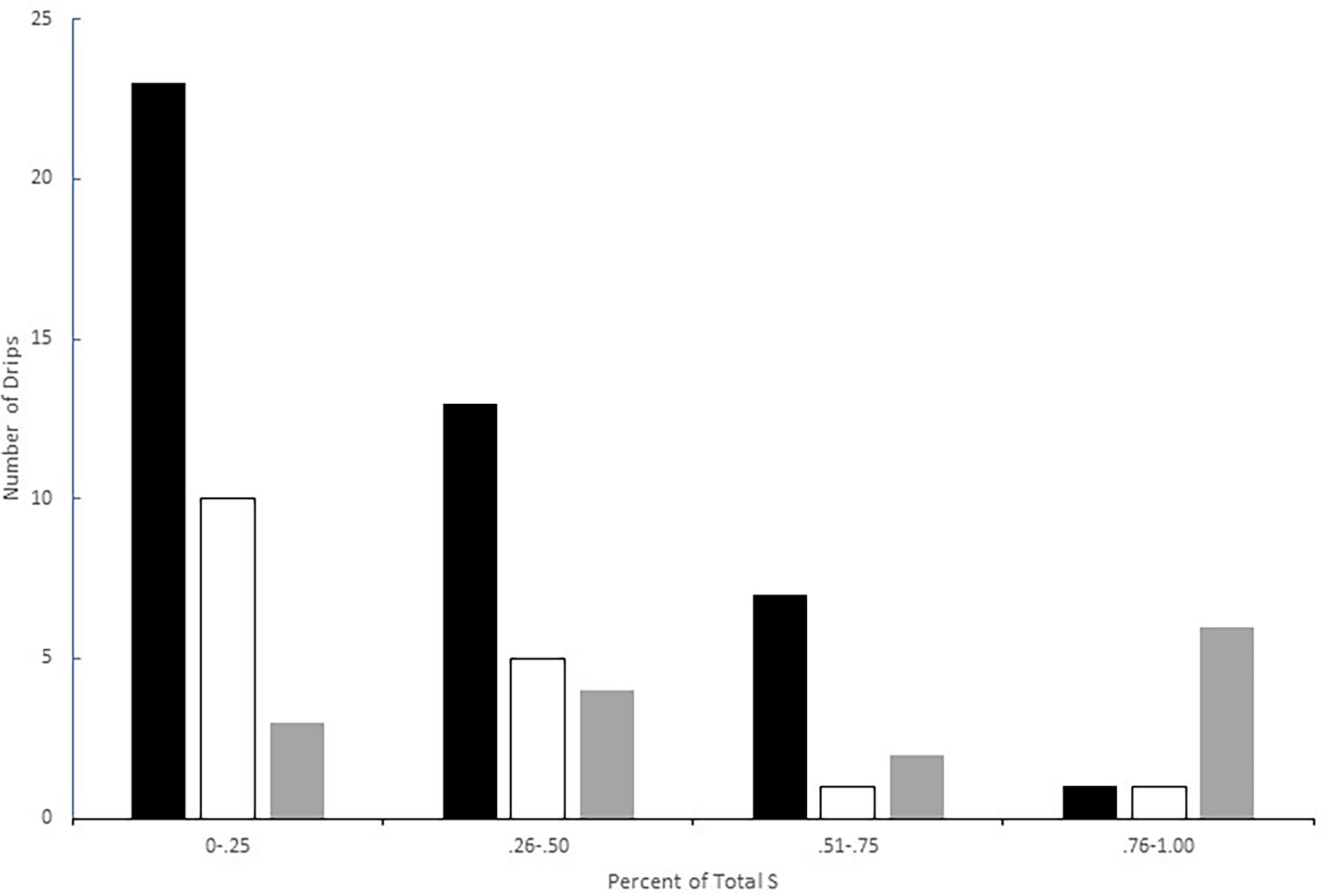
Histogram of the number of copepod species per drip scaled to the total number copepod species found in the cave where the drip is located. Black bars are the Dinaric Karst, white bars the Isolated Karst, and gray bars the Alpine Karst. Velika pasica was not included because of much longer sampling times (Table 1).

The distribution of number of epikarst copepod species per drip, scaled by the total number of epikarst copepod species in the cave provides a visualization of the partitioning of species richness (Fig 2). The primary difference between species rich and species poor caves is not evidenced in all drips but rather in a small number of drips with most of the copepod species known from the cave (Table 4 and Fig 2). For example, in Dimnice, three drips have less than three species, and its high species richness (eight species) is determined largely by two drips. In Županova jama, the cave with the most stygobiotic copepods among the sampled caves, one drip contained 10 of the 13 species known, and one drip had only one species. The exception is the Alpine karst, where 40% of the drips contain all the species. However, no more than three species were known from any Alpine cave (Table 2). Most drips (71 of 81) have between zero and four species, regardless of the cave or region.

Mean drip species richness relative to the total number of species found in its quadrat (cave) ranged from 0.12 to 0.71 (Table 5). Not surprisingly, mean species drip number and total species number were more similar in low diversity caves. The ratio of maximum single drip species number and total cave species richness ranged from 1 to 0.50. The maximum number of species in an individual drip in a cave was a better predictor of total species number in that cave than was mean species number in a drip (R^2^_adj_ = 0.92 compared to R^2^_adj_ = 0.75). Sorenson dissimilarity ranged from 0 in Snežna jama to 0.87 in Postojnska jama. Except for low richness caves in Alpine karst, turnover, even at this small scale, accounted for over 60 percent of β-diversity, except for Velika pasica, which was sampled for multiple years.

**Table 5:** Minimum, maximum, and mean number of stygobiotic copepod species (S) per drip (S_d_), and total cave species richness. Max S_d_/S is the ratio of species numbers in the richest drip to the cave total; mean S_d_/S is the ratio of mean species numbers in drips to the cave total. Β_sor_ is Soresnson dissimilarity, and % turnover is the contribution of turnover to Sorenson dissimilarity [37].

Cave	Min S_d_	Max S_d_	Mean S_d_	Total Cave S	Max S_d_/S	Mean S_d_/S	β_Sor_	% due to turnover
**Dinaric karst**								
Črna jama	2	6	3.6	8	0.75	0.45	0.66	81
Dimnice	1	7	3.2	8	0.88	0.40	0.76	66
Pivka jama	2	6	3.2	8	0.75	0.40	0.57	62
Postojnska jama	0	2	0.5	4	0.50	0.13	0.87	96
Škocjanske jame	1	5	3	8	0.63	0.38	0.73	84
Velika pasica	4	6	4	7	0.86	0.57	0.32	42
Zguba jama	0	4	1.4	6	0.67	0.23	0.59	74
Županova jama	1	10	5	13	0.77	0.38	0.77	38
**Alpine karst**								
Jama pod Babjim zobom	0	3	1.6	3	1.00	0.53	0.44	0
Pološka jama	1	3	1.8	3	1.00	0.60	0.45	0
Snežna jama	0	2	0.8	2	1.00	0.40	0	
**Isolated karst**								
Zadlaška jama	0	2	0.8	3	0.67	0.27	0.75	89
Huda luknja	0	2	0.33	2	1.00	0.17	0.54	74

At the regional scale, Sorenson dissimilarity was highest in the Isolated karst, not surprisingly since the two caves are quite far apart (Fig 1). The percent contribution of turnover was very high at the regional scale, ranging from 88 to 100% (Table 6).

**Table 6:** Comparison of observed and estimated total epikarst copepod species richness for the Alpine karst, Isolated karst, and the Dinaric karst. When the coefficient of variation for incidence based distribution is greater than 0.5, as it is in this case, Chao [37] recommends using classic rather than unbiased estimators for Chao2, and using the larger of Chao2 and ICE estimates. Both are shown below. Sorenson’s dissimilarity index is also shown, as is the percent contribution of turnover to this dissimilarity [36].

Region	S	Chao2	ICE	N of caves	Β_Sor_	% turnover
Alpine	6	6.0	10.9	3	0.57	88
Dinaric	25	40.4	41.4	8	0.76	88
Isolated	4	7.0	7.0	2	1.00	100

### Accumulation curves

**Fig 3:**
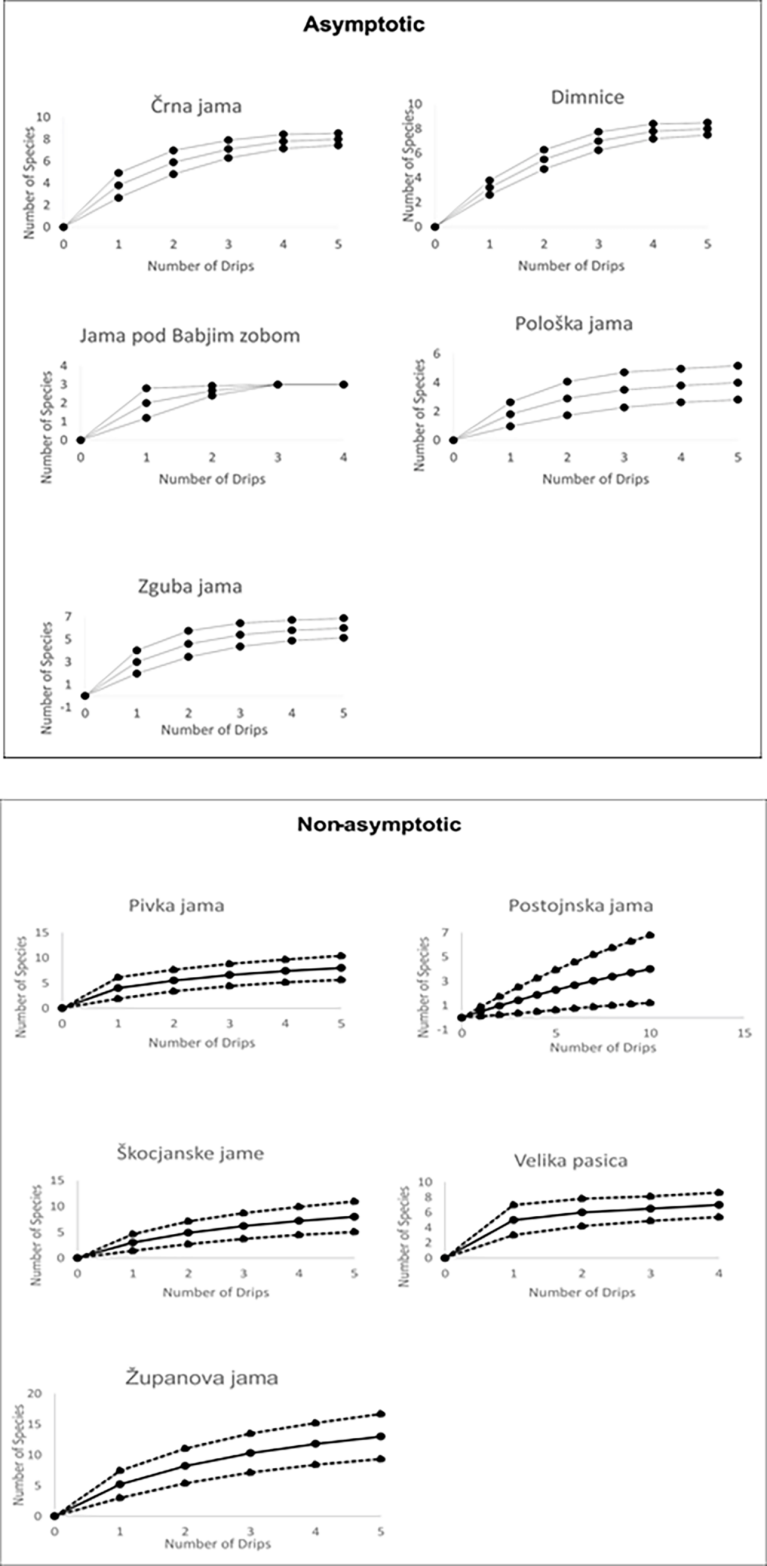
Species accumulation curves for stygobiotic epikarst copepods in Slovenian caves. Top panel: Asymptotic curves; Bottom panel: Non-asymptotic curves. Upper and lower curves are the 95 percent confidence intervals. Curves were created in EstimateS [18].

Unlike the Chao1 estimates, which indicated sampling completeness except for Škocjanske jame, accumulation curves for four caves in addition to Škocjanske jame indicate incomplete sampling—Postojnska jama, Pivka jama, Velika pasica, and Županova jama, by a criteria of at least 0.5 species difference between the last and next to last species estimates from the accumulation curves (Fig 3). Two caves—Huda luknja and Snežna jama—only had two species and so there were insufficient data for this analysis.

Both Chao2 and ICE estimates (Table 6), indicate that sampling is very incomplete for all areas. This is not surprising given the high level of endemism seen in the data (Table 2).

**Fig 4:**
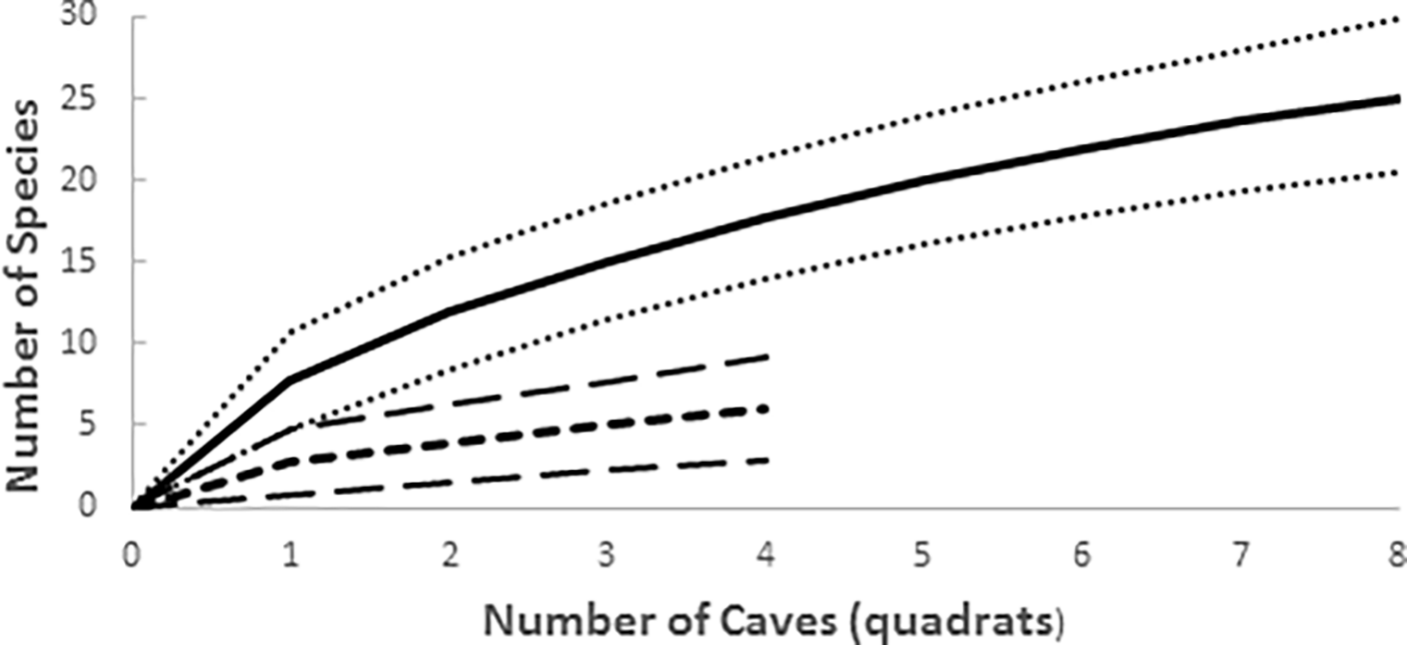
Accumulation curves for epikarst copepod species in the Alpine (dotted line) and Dinaric (solid line) karst. Since only two caves were sampled in the Isolated karst, it is not included.

Because sampling at the regional scale is incomplete, estimates of species richness are sensitive to the number of caves (quadrats) sampled. Therefore, the two regions are compared at n = 3 caves of their respective accumulation curves (see [37]). Isolated karst was not included because there were only two sampled caves in this region. The curves do not cross so the differences may be general (Fig 4). In the Alpine karst, mean cave (quadrat) species richness is 54 percent of the four cave average, while in the Dinaric karst it is 52 percent. In the Alpine karst, maximum species richness (3 in Jama pod Babjim zobom and Pološka jama) is 50 percent that of regional species richness. In the Dinaric karst, maximum cave species richness (13 in Županova jama) is 86 percent of regional richness in the Dinaric karst.

### Partitioning of species diversity

**Fig 5:**
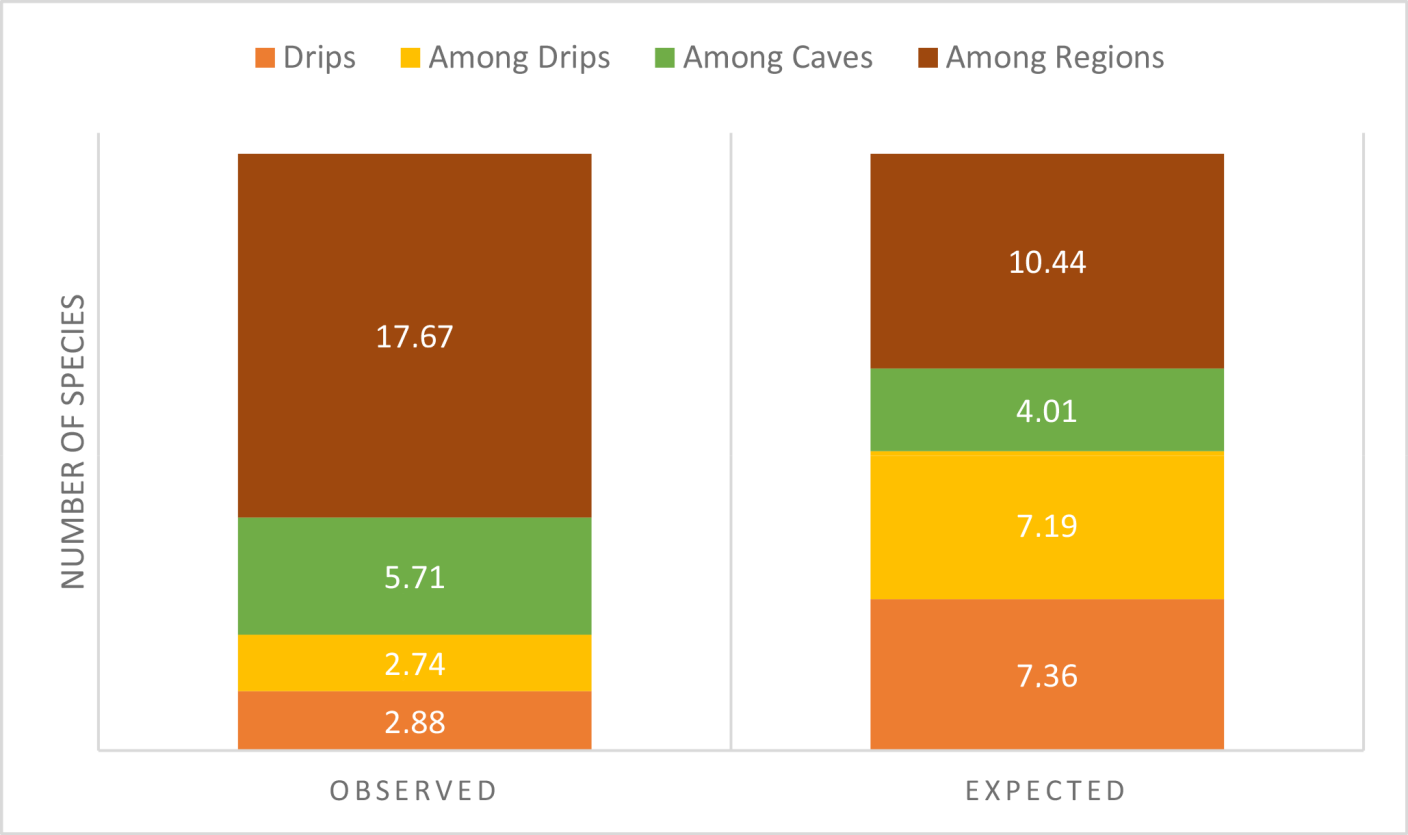
Relative contribution of within drip (α-diversity), among drip, among cave, and among region diversity (all β-diversity) to the overall diversity of 30 epikarst copepod species, relative to random expectation.

Slightly more than 90% of observed species richness is β-diversity, with the bulk of the β-diversity being between regions (63%, Fig 5). Relative to expected values generated by 1000 randomizations of individuals samples [38], both observed α-diversity and between drip β-diversity are reduced. When the relative importance of replacement and nestedness was compared, replacement accounted for 66% and nestedness accounted for 34% of β-diversity.

## Discussion

### Geographic pattern

At the regional scale, species richness is highest in the Dinaric karst, relative to both the Alpine and Isolated karst. This holds for the observed number of epikarst stygobiotic copepods known from each region and the Chao 2 and ICE estimates for each region, as well as the differences in species accumulation curves between the Dinaric and Alpine karst. With the exception of Postojnska jama, all caves in the Dinaric karst had at least six species, double that of any other cave studied.

When individual drips are considered, the clear pattern of uniformly high species richness in the Dinaric karst begins to blur. If only the single most species-rich drips in each cave are compared, the overall pattern (and relative species richness) of both caves and regions is apparent. When minimum values of species numbers in a drip is considered, the differences among regions breaks down entirely. The importance of a few species rich drips is also reflected in the importance of nestedness for β-diversity. While replacement is still more important, nestedness makes a significant contribution, both at the scale of drips and the scale of caves, unlike the case of the large scale pattern (2500 km^2^ quadrats) where nestedness accounts for only about 5% of β-diversity [38]. This pattern of a small proportion of very rich drips has been reported for other epikarst studies from West Virginia, USA [39] and Romania [40].

The heterogeneity of the pattern of species richness among caves and particularly among drips within a cave is difficult to explain. If there is an abundance of explanations for regional differences, there is a shortage of credible explanations for the fine scale pattern. In the study of the epikarst fauna of Črna jama, Dimnice, Pivka jama, Postojnska jama, Škocjanske jame, and Županova jama, chemical differences between the water in different caves, and in the niches of different copepod species were found [19,32,41], but the high diversity drips themselves do not stand out in any way. There are likely differences in the pathways and retention time of water in different drips, a result strongly implied by hydrological analyses [23], and measurements of age of the water, by tritium [42] or other methods would be most instructive in this regard. Likewise, long-term measurements of drip rate [23,43] by hydrologists should yield biological insights when done on biologically interesting and sampled drips. These hydrological studies show considerable variation among drips in their connection to water reservoirs, and proportion of diffuse and fracture flow [44].

One physical variable that may be an explanatory variable is ceiling thickness. One of the first sites where a high diversity of epikarst copepods was found was Velika pasica [45], a small, shallow cave. Županova jama, the most species rich cave in this study, also has a thin ceiling, and Postojnska jama, a species poor cave, has a thick ceiling [46]. This result may seem paradoxical because a thinner ceiling (overburden) would seem to mean less available habitat. The habitat above a cave passage (the percolation or unsaturated zone), has two components—a zone of vertically moving percolating water and a storage zone, the epikarst. Williams [21] reviews the extensive hydrogeological evidence for the existence of epikarst, a widely [47] but not universally held view [48]. A thin ceiling means a shorter and smaller percolation zone, which may act as a filter for epikarst species that get dislodged from the epikarst, and it also means a shorter distance to the soil, a source of organic carbon to epikarst [49]. The demonstration that the occurrence of all but one of 23 stygobiotic epikarst copepods in six Slovenian caves was negatively correlated with ceiling thickness [41,50] lends credence to this view. Epikarst species do also occur in the percolation zone [51], but almost certainly in diminished numbers. Drip pools in caves, part of the percolation zone, contain elements of the epikarst fauna but also contain non-epikarst, non-specialized species [27]. In spite of the apparent importance of ceiling thickness, it cannot explain the pattern of differences among caves in the Dinaric karst reported in this study. This is highlighted by Zguba jama, a very shallow cave, which was chosen for study in part because it is so shallow, has an unremarkable fauna. In addition, Škocjanske jame, thick-ceilinged caves, have a rich fauna.

### Epikarst–cave comparisons

Subterranean biologists have been slow to summarize species richness and diversity patterns, both because of the general difficulty in sampling caves and the recognition that high levels of endemism [52] result in incomplete species lists. Sket [53,54] was among the first to tackle this problem, and focused on the Dinaric karst and its subterranean biodiversity in Slovenia [16, 32, 55, 56]. A notable exception to this neglect of the Alpine and Isolated zones is Novak’s work on the terrestrial cave fauna of the Isolated karst [57]. Sket et al. [58] also touch on the non-Dinaric fauna of Slovenia in their review of the obligate subterranean fauna of the Balkans. As far as we can determine, ours is the first study to quantitatively compare species richness in the three regions of Slovenia.

The explanation of high epikarst species richness in the Dinaric region relative to the Alpine and Isolated regions is likely several-fold. Temperate zone high elevation karst areas are not rich in obligate subterranean species because of low surface productivity, upon which the subterranean communities ultimately depend, as well as low ambient temperatures. In some cases, the epikarst zone may be frozen, at least part of the year, which may explain the absence of any copepods from some drips in caves such as Snežna jama (Table 4). While both the Alpine and Dinaric karst regions are more or less contiguous, the Isolated karst is dissected and island-like, perhaps resulting in a reduction in species richness, a general characteristic of island-like habitats [59].

The other half of the explanation of the high species richness of the Dinaric karst epikarst fauna is the reason why the Dinaric karst is a global hotspot of subterranean species richness in general. A number of explanations have been put forward for this [10], including high density of caves and amount of karst [11,55], high productivity [11], proximity to the sea and enhanced opportunities for invasion [53], proximity to groundwater [60], and the long and complex geological history of the region [53]. There is much less information available on epikarst species richness outside Slovenia, with the exception of Romania [40,61]. For five Romanian caves, mean number of stygobiotic copepods per cave was 4.8, with a total Chao2 estimate of species richness of 15.5 [50]. This puts it lower than the Dinaric karst both for mean cave and regional species numbers, but higher than Alpine and Isolated regions in Slovenia. Eme et al. [62] argue that there is no one single reason for high subterranean crustacean diversity and elsewhere along the so-called ridge of high species richness [11], which seems to hold for both aquatic and terrestrial subterranean species. Eme et al. [62] demonstrate the general importance of spatial non-stationarity, and show that both spatial heterogeneity and productive energy are important to the south while historical climate stability was important to the north.

Within the Dinaric karst, there is no relationship, at the level of individual cave, between epikarst hotspots and hotspots for other components of the subterranean fauna. Many of the single cave hotspots for the non-epikarst fauna (*e*.*g*., Lukova jama pri Zdihovem, Logarček, Križna jama, Mačkovica, Predjamski sistem, and Šica-Krka sistem) have not been sampled for epikarst fauna and no comparison is possible. However, in the case of the epikarst fauna sampled in the eight caves in the Dinaric karst in this study, comparison with other parts of the subterranean fauna can be made. For epikarst fauna, there are two cave hotspots—Škocjanske jame and Županova jama. Neither of these caves is a global hotspot of cave biodiversity, although a number of caves in the Dinaric karst are, including Postojna Planina Cave System, which including Postojnska jama, Pivka jama, and Črna jama [12,60]), and neither Škocjanske jame nor Županova jama is a regional cave hotspot of terrestrial diversity, although Dimnice and the Postojna-Planina Cave System are [63].

### Partitioning species diversity

The species diversity patterns are the result of using the individual drip samples as replicates for each cave (or more properly, each 1 km^2^ quadrat), but each individual drip actually drains a separate miniature subsurface basin [64], which may differ among themselves in terms of area drained and response time to precipitation events [23]. A single drip is the outlet of a miniature drainage basin. Typical subsurface drainage basins emerging in karst springs are tens to hundreds of square kilometers in size [65], while the calculated area of three epikarst drips ranged less than 1 m^2^ to slightly more than 200 m^2^ [23]. At this very small scale, the average species richness in a drip contributed 10 percent (three species) of total regional species richness overall. Caves, corresponding to a 1 km^2^ quadrat, contributed approximately an additional 30 percent of total species diversity in all the regions. This is not so different from the results of Malard et al. [8] and Eme et al. [62] for the European groundwater fauna, except that the geographic scale for epikarst copepods is reduced by an order of magnitude.

When the data are viewed in another way, one that emphasizes the occurrence of “hotspot” drips, a different pattern emerges. A few drips contribute a disproportionate share of species diversity. The maximum species rich drip in the Dinaric karst has 10 species and occurs in Županova jama, and Županova jama itself has 13 species, so this drip contributes 40 percent of the species diversity known from the entire Dinaric karst! The task of assessing epikarst species diversity would be considerably simplified if we had a method of determining which drips were hotspots prior to sampling, but we don’t.

If indeed the pattern of epikarst species diversity is one of regional differences but the result of a few hotspots, perhaps about 10 percent of sampled drips, then accumulation curves may be misleading. They measure the probability of including a hotspot drip, rather than a sample of similar drips all of which may contain all the species (see [66] for a similar problem). This is not a suggestion to abandon accumulation curves, but rather to also consider that there is some unmeasured fine-scale difference that is important.

## Conclusions

As is the case with other obligate subterranean communities, β-diversity is much greater than α-diversity in epikarst communities, as a result of differences on a fine scale, for example, differences among drips in a cave, typically only a few tens of meters apart contribute to diversity. While the replacement component of β-diversity predominates, as it does in other subterranean communities, the nestedness component is also important. It manifests itself in the form of a small number of hotspot drips, and a relatively small number of drips largely determine overall species diversity.

These fine-scale differences are also relevant to any fauna protection plan so that small hotspots are not ignored. While it is tempting to focus on the individual drip and the drip pool beneath it (if one is present), it is not the drip pool but the overlying epikarst that is the critical habitat. The pool is typically a subsample of the epikarst fauna, with less specialized elements present as well [27]. Because the epikarst is typically shallow (only a few meters in depth [21–23]), the focus of any successful epikarst protection plan should be the protection of the surface landscape and processes.

## Supporting information

S1 Table Numbers of copepods found, by species and by drip, in the study caves of the Dinaric region. (XLSX)

S2 Table Number of copepods found, by species and by drip, in the study caves of the Isolated region. (XLSX)

S3 Table Number of copepods found, by species and by drip, in the study caves of the Alpine region. (XLSX)
